# Using Cardiovascular Cells from Human Pluripotent Stem Cells for COVID-19 Research: Why the Heart Fails

**DOI:** 10.1016/j.stemcr.2020.11.003

**Published:** 2020-12-10

**Authors:** Loukia Yiangou, Richard P. Davis, Christine L. Mummery

**Affiliations:** 1 Department of Anatomy and Embryology, Leiden University Medical Center, Einthovenweg 20, 2333 ZC Leiden, the Netherlands

**Keywords:** SARS-CoV-2, COVID-19, hPSCs, cardiovascular cells, disease modeling, drug screening, myocardial injury, arrhythmias, cytokine storm

## Abstract

The severe acute respiratory syndrome coronavirus 2 (SARS-CoV-2) led to the coronavirus disease (COVID-19) outbreak that became a pandemic in 2020, causing more than 30 million infections and 1 million deaths to date. As the scientific community has looked for vaccines and drugs to treat or eliminate the virus, unexpected features of the disease have emerged. Apart from respiratory complications, cardiovascular disease has emerged as a major indicator of poor prognosis in COVID-19. It has therefore become of utmost importance to understand how SARS-CoV-2 damages the heart. Human pluripotent stem cell (hPSC) cardiovascular derivatives were rapidly recognized as an invaluable tool to address this, not least because one of the major receptors for the virus is not recognized by SARS-CoV-2 in mice. Here, we outline how hPSC-derived cardiovascular cells have been utilized to study COVID-19, and their potential for further understanding the cardiac pathology and in therapeutic development.

## Teaser

In this perspective, Mummery and colleagues outline how hPSC-derived cardiovascular cells can be used to investigate COVID-19. They describe the recent advances these models have provided in uncovering how SARS-CoV-2 infects the heart and propose how they could be further utilized to rapidly advance our understanding of the resulting cardiovascular pathology, as well as their potential role in developing therapies.

## Main Text

### Introduction

Coronavirus disease (COVID-19) is caused by severe acute respiratory syndrome coronavirus 2 (SARS-CoV-2) viral infection, and understanding how this deadly virus infects and affects humans is currently under intense investigation. Knowledge of this new virus has evolved in the past 9 months, while valuable information on its pathology in humans has also been derived from previous studies of closely related viruses. SARS-CoV and MERS-CoV, which led to the SARS and Middle East Respiratory Syndrome (MERS) outbreaks, respectively, provided essential basic information on the molecular structure, genome, and virus mechanisms for host cell entry (Wu et al., 2020; Zhou et al., 2020b). Moreover, clinical data from COVID-19 patients have shed light on various aspects of the pathology (Geng et al., 2020). To date, there are no vaccines, and the effectiveness of the few treatments available is highly dependent on the patient's condition when administered. This highlights the urgency for relevant human studies, in particular due to COVID-19 affecting individuals to strikingly variable extents. Whereas the majority of individuals experience little more than common cold or flu-like symptoms, others experience severe symptoms culminating in death. Epidemiological and clinical studies have associated these variable outcomes of infection with gender, age, and obesity (Richardson et al., 2020), but there are still many unknown factors. In addition, variability in COVID-19 severity also depends on underlying and pre-existing co-morbidities such as diabetes mellitus, chronic pulmonary disease, cancer, hypertension, and cardiovascular disease (CVD). Although the dominant pathology involves the respiratory system, COVID-19 also causes cardiovascular complications, namely myocardial injury, arrhythmias, acute coronary syndrome, and vascular damage, including thromboembolism (Nishiga et al., 2020; Zheng et al., 2020). Indeed, clinical data to date suggest that 20%–30% of COVID-19 patients experience severe cardiovascular damage, which significantly contributes to poor prognosis (Huang et al., 2020; Nishiga et al., 2020). Increased troponin I expression, widely used as a biomarker of myocardial infarction, is among the best predictors of those patients who will die in intensive care (IC) (Guo et al., 2020; Huang et al., 2020; Tersalvi et al., 2020). Moreover, patients with pre-existing cardiovascular conditions are more likely to develop severe illness and have higher risk of death compared with patients without co-morbidities (Huang et al., 2020). These detrimental effects on the heart highlight the need for better understanding of the molecular basis of cardiac damage caused by SARS-CoV-2, with a view to developing more effective therapeutics to prevent this. Specific questions include: (1) which of the multiple cell types in the heart are SARS-CoV-2 targets, and how are these cells affected; (2) what is the role of the inflammatory response; and (3) do COVID-19-associated thrombi in the vasculature cause micromyocardial infarctions?

Most studies on SARS-CoV-2 and other closely related viruses use either genetically modified mouse models (Bao et al., 2020) or immortalized cell lines, such as Vero cells derived from the African green monkey or human cancer cell lines (Chu et al., 2020; Hoffmann et al., 2020; Ou et al., 2020; Shang et al., 2020). Although these cell lines possibly reflect virus entry and/or sustain viral replication, they miss the tissue-specific physiology necessary to understand why some organs are affected different from others. Investigating the pathology of the disease using animal models has also been a challenge. In humans, the major receptor used by SARS-CoV-2 to enter cells is angiotensin-converting enzyme 2 (ACE2). However, the ortholog Ace2 receptor in mice does not bind SARS-CoV-2. Thus, for mice to be used as a model organism to study SARS-CoV-2, humanized mouse models have been generated in which the human ACE2 receptor is overexpressed (Bao et al., 2020; Sun et al., 2020). This can provide information on the initial stages of the virus life cycle and cell entry and support studies on antiviral drugs for blocking viral entry. However, human pathology following SARS-CoV-2 infection is not entirely captured by these models, either. For example, it has been reported that, unlike in humans, where the disease is principally a respiratory illness, SARS-CoV-2 infection in mice primarily involves the nervous system (Sun et al., 2020). These differences in host receptor expression and infection modalities highlight key interspecies differences in virus tropism. There are also reports of cats, dogs, ferrets, minks, and hamsters becoming infected by SARS-CoV-2, with some of these animals reflecting the respiratory effects observed in humans (Shi et al., 2020). Although these animals appear to closely reflect the human pathology, they are not readily accessible for routine research use.

As an alternative to immortalized human cell lines or model organisms, human pluripotent stem cells (hPSCs) are also regarded as relevant *in vitro* models to study COVID-19, and most particularly for the effects of SARS-CoV-2 infection or antiviral drugs on the heart, since there are no alternative *in vitro* human cardiac models other than primary tissue. hPSCs include both human embryonic stem cells (hESCs) and human induced PSCs (hiPSCs) generated by reprogramming somatic cells (Takahashi et al., 2007). Over the past 2 decades, numerous protocols have been established that allow successful generation of cardiac-derived cell types, namely cardiomyocytes (CMs), endothelial cells (ECs), cardiac fibroblasts (CFs), smooth muscle cells (SMCs), and pericytes (Matsa et al., 2016; Paik et al., 2020; Passier et al., 2016; Samak and Hinkel, 2019). Moreover, protocols using hPSCs to generate other cell types found in the heart (such as macrophages) have also been developed (Cao et al., 2019; Lee et al., 2018; Montel-Hagen and Crooks, 2019). These protocols follow developmental principles with the cells used for multiple applications, ranging from modeling cardiovascular development and disease to drug screening studies to identify novel therapeutic agents or test drug cytotoxicity (Brandão et al., 2017; Denning et al., 2016; Protze et al., 2019). Many of these cell types can also be cryopreserved, thus providing a readily accessible platform for such studies (van den Brink et al., 2020; Giacomelli et al., 2020). Furthermore, hPSC-derived cell types have been used to model and investigate infectious diseases (Rowe and Daley, 2019), demonstrating their potential suitability for studying COVID-19.

**Figure 1:**
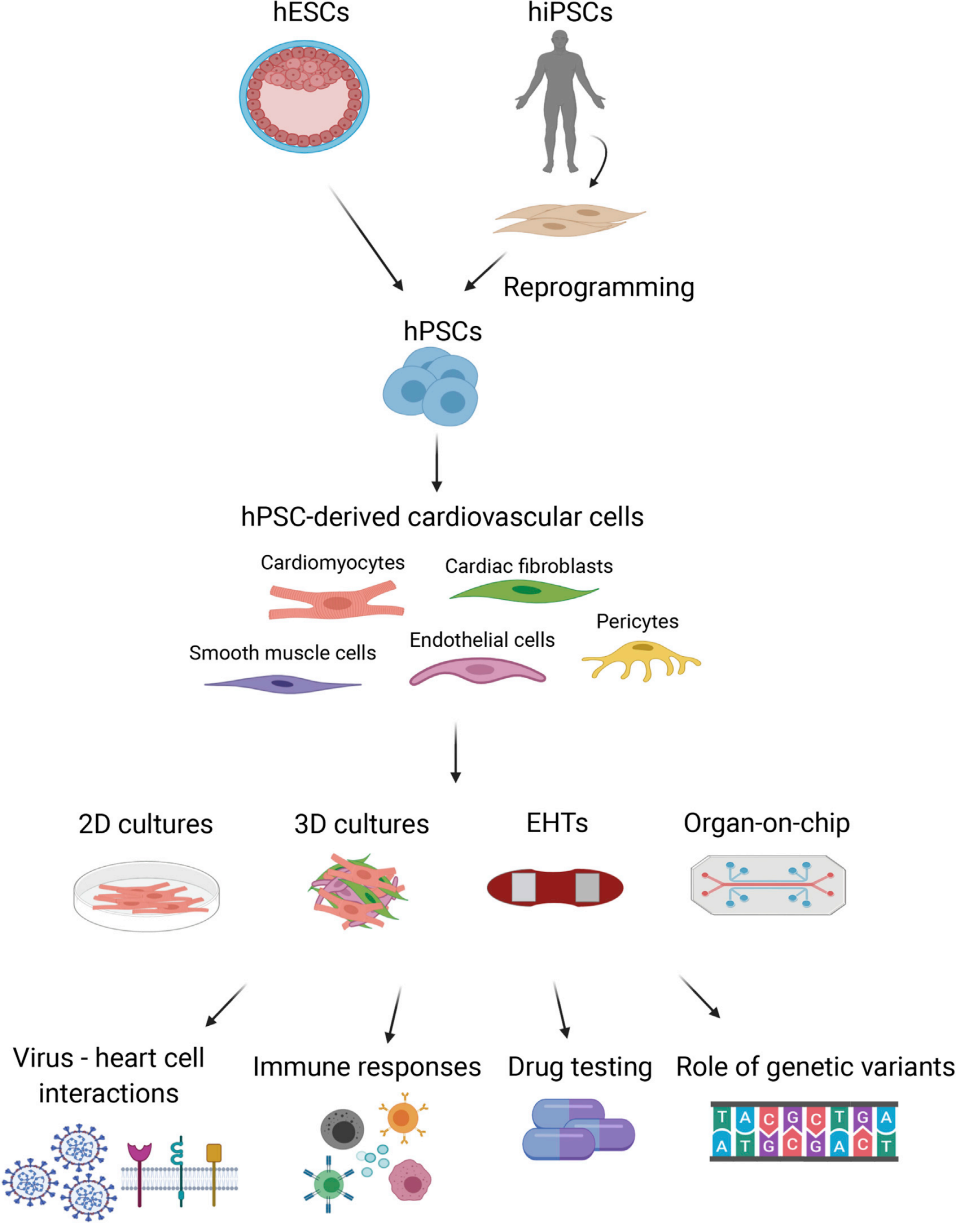
Overview of Potential Applications of hPSC-derived Cardiovascular Cells for COVID-19 Research The hESCs or hiPSCs can be used to generate various cardiovascular cell types *in vitro*, including cardiomyocytes, fibroblasts, endothelial and smooth muscle cells, and pericytes. The resulting cells can be used in assays in a variety of formats, such as 2D monolayers, 3D multicellular cultures, EHTs, or culture on organ-on-chip devices. Subsequently, various biochemical, cellular, molecular, and genetic studies can be performed to assess virus-host interactions or immune responses, for drug screening, or to examine the role of particular genetic variants in disease susceptibility.

First, the cellular and molecular host-virus interactions can be examined, specifically, the mechanisms through which cardiovascular cells are infected by SARS-CoV-2 and potentially the identification of long-term effects of the infection on recovered patients. Second, they can provide a preclinical platform for COVID-19 drug testing. Repurposed drugs can be screened for potential effectiveness in combating COVID-19, while novel drug candidates can be examined for cardiac cytotoxicity, a possible secondary risk for COVID-19 patients. This can be investigated using 2D monolayer cultures of hPSC-derived CMs (hPSC-CMs), as well as 3D multicellular systems such as cardiac microtissues (Giacomelli et al., 2020), engineered heart tissues (EHTs) (Eschenhagen et al., 2012; Mannhardt et al., 2016), or heart-on-chip models (Jastrzebska et al., 2016; Ribas et al., 2016). These cellular models can be adapted to answer specific SARS-CoV-2-related biological questions (Figure 1). In this perspective, we discuss how hPSC-derived cardiovascular cells are being used to understand the effect of SARS-CoV-2 on the heart and how they can be further utilized for the development of therapeutics for COVID-19.

### SARS-CoV-2 Interactions with the Heart

#### Structure and Cell Entry Mechanism of SARS-CoV-2

SARS-CoV-2 is composed of four structural proteins, namely the spike (S), envelope, membrane, and nucleocapsid proteins, and a 30-kb positive-sense, single-stranded RNA genome (Wang et al., 2020d). The virus uses the S protein to bind to and enter human cells. The first receptor identified to be bound by SARS-CoV-2 was ACE2 (Li et al., 2003), a membrane-localized aminopeptidase that is highly expressed in the heart, blood vessels, kidneys, and lungs and has a vital role in the cardiovascular and immune systems (Turner et al., 2004). Following binding, the S protein is cleaved by the transmembrane serine protease TMPRSS2, a process known as S-protein priming. This facilitates the entry of the virus into the host cell cytoplasm by direct fusion with the cell membrane and is thought to be the predominant initial *in vivo* mode of entry through respiratory tract epithelial cells. The heart expresses high levels of ACE2 too, and while this receptor plays a clear role in the key cardiac pathologies developed in COVID-19, the exact mechanisms of how ACE2 interaction with the virus leads to the observed pathology in patients are elusive (Bansal, 2020; Zheng et al., 2020). Evidence from mouse models of SARS suggests that ACE2 levels are reduced upon infection, with ACE2 knockout resulting in reduced cardiac contractility (Nishiga et al., 2020); however, whether this occurs in COVID-19 is unknown. Moreover, although ACE2 is highly expressed in human CMs and pericytes (Chen et al., 2020), expression of TMPRSS2 appears absent (Litviňuková et al., 2020), highlighting the possibility that alternative mechanisms may facilitate viral entry of SARS-CoV-2 into the human heart. Indeed, a recent preprint study using hPSC-CMs confirmed TMPRSS2 expression was not detectable, and demonstrated that SARS-CoV-2 could infect these CMs via an endolysosomal route, specifically via cathepsin-L protease (Pérez-Bermejo et al., 2020). Whether this is the predominant mode of infection of CMs requires further investigation, but highlights that different treatment strategies may be needed for treating different organs.

The transmembrane protein CD147, also known as Basigin or extracellular matrix metalloproteinase inducer (EMMPRIN), has also been proposed as a potential target in COVID-19 treatments. This protein is utilized by the parasite *Plasmodium falciparum*, which causes malaria in humans (Crosnier et al., 2011), and a few studies demonstrated that it serves as an alternative receptor that can be bound by SARS-CoV (De Wit et al., 2016). Whether CD147 also plays a role in SARS-CoV-2 entry is unclear, with one preprint study demonstrating that it could be bound by SARS-CoV-2 (Wang et al., 2020b), while a second report failed to show interaction of the virus S protein with CD147 expressed on human cells (Shilts and Wright, 2020). Thus, the role of CD147, as well as its possible therapeutic impact, requires further validation, with hPSC-derived cell types potentially facilitating this effort. Moreover, a third transmembrane protein identified to be bound by SARS-CoV-2 is neuropilin-1 (Cantuti-Castelvetri et al., 2020; Daly et al., 2020). Neuropilin-1 is an important co-receptor expressed in the cardiovascular system, playing roles in angiogenesis, axon guidance, cell survival, migration, and invasion. How SARS-CoV-2 binds to this protein and the resulting downstream effects, especially in the heart, are currently unknown; however, NRP-1-deficient mice develop cardiomyopathy and aggravated myocardial infarction-induced heart failure.

Therefore, hPSC-derived cardiovascular cells will likely provide a useful and physiologically relevant platform to identify which membrane-localized proteins in the heart are essential for SARS-CoV-2 entry and how the virus interacts with them to facilitate this, as well as the cardiovascular cell types expressing these receptors. Since the SARS-CoV-2 outbreak, a few studies have utilized hPSC-derived heart models to address these questions. These studies have collectively reported that the ACE2 receptor is expressed in mesodermal derivatives, namely macrophages, ECs, and CMs (Marchiano et al., 2020; Pérez-Bermejo et al., 2020; Yang et al., 2020). Furthermore, hiPSC-CMs have been shown to be susceptible to SARS-CoV-2 infection and further viral replication (Marchiano et al., 2020; Pérez-Bermejo et al., 2020; Sharma et al., 2020), demonstrating their suitability for studying the resulting downstream effects and mechanisms.

Similarly, hiPSC-derived blood vessel organoids can also be directly infected by SARS-CoV-2, with human recombinant soluble ACE2 blocking entry of the virus (Monteil et al., 2020). Interestingly, while ECs express ACE2 (Hamming et al., 2004), they were not infected by the virus in two recent studies of hiPSC-ECs (Pérez-Bermejo et al., 2020; Yang et al., 2020). However, a number of clinical studies have shown damage of the endothelium caused by SARS-CoV-2 (Huertas et al., 2020; Varga et al., 2020), leaving open questions requiring further investigation. For example, are these discrepancies linked to the developmental origin of the ECs or the maturation state of the hPSC-derived ECs. Despite not appearing to be infected, hiPSC-ECs exposed to SARS-CoV-2 did exhibit significant cytopathic effects (Pérez-Bermejo et al., 2020), raising the possibility that the endothelial injury observed in COVID-19 patients might not be directly due to viral infection of the ECs but occurs via paracrine effects instead. Further research to determine whether the interaction of SARS-CoV-2 with ECs is accurately reflected in hPSC-EC models is still necessary.

The heart also comprises a significant fraction of CFs, currently estimated to be about 15%. Their important role in the myocardial response to injury makes them also a potential key cell type in COVID-19 pathology. Like hiPSC-ECs, although the ACE2 transcript was not detected in hiPSC-CFs and they did not appear to be infected by SARS-CoV-2, toxicity was still observed following exposure to the virus (Pérez-Bermejo et al., 2020). Further investigation into the mechanism causing this response and whether they might still play a role regarding infection and propagation of the virus when present in a multicellular structure is required. Mural cells such as vascular SMCs and pericytes also express ACE2 (Chen et al., 2020; Hamming et al., 2004; He et al., 2020); however, the tropism of SARS-CoV-2 for these two cell types is currently unknown. The important role of these cells in vasculature homeostasis indicates that their potential infection could lead to the adverse vascular damage and inflammation seen in COVID-19 patients. This highlights the importance of precisely identifying not only the cell types in the heart that are readily infected by SARS-CoV-2, but also how the virus affects the other cell types present and whether this contributes to the pathology observed in COVID-19 patients.

Further studies need to also address the role of neuropilin-1 in SARS-CoV-2 infection. Already available transcriptomic data indicate that this gene also is expressed in hiPSC-derived cardiac ECs and CFs. However, further analysis is required to determine its expression at the protein level in these cells, as well as downstream biological processes that could be activated following interaction of this protein with the virus.

#### Cardiovascular Damage in COVID-19 Patients

COVID-19 patients often present with multiple cardiovascular complications. Pathological findings in the hearts of COVID-19 patients have provided useful insight into these complications and laid the basis for determining aspects of the disease that need modeling. COVID-19 patients admitted to IC die predominantly from respiratory failure, septic shock and multi-organ failure, or cardiac failure (Bansal, 2020; Huang et al., 2020). Recent reports have shown that the majority of these patients also suffer from acute cardiac injury, evidenced by increased levels of serum troponin I (Huang et al., 2020; Lippi and Plebani, 2020). Indeed, elevated troponin I levels distinguish those patients with severe/fatal disease from those who are eventually discharged from IC (Huang et al., 2020; Wang et al., 2020a). Another indication of myocardial injury in COVID-19 patients is arrhythmias, which may also be fatal (Wang et al., 2020a). Poor prognosis of patients with cardiac injury or heart failure highlights the key role that heart damage plays in the lethality of the disease. However, it is unclear whether the severe cardiovascular effects resulting from SARS-CoV-2 infection are caused by direct virus-induced myocardial injury, indirect systemic effects such as inflammation, or a combination of both.

Thus, hPSC-derived cardiovascular cells will likely provide a valuable tool to model *in vitro* the events leading to cardiovascular damage observed in COVID-19 patients. Myocardial injury, as it manifests in humans, can be confirmed by comparing troponin I levels in infected and uninfected cardiac cells. Apart from evaluating the myocardial injury itself, key mechanisms that drive it can also be investigated in depth using hPSC model systems, following either direct infection of the myocardium or activation of inflammatory responses. A number of reports and preprints have shown that viral infection of hPSC-CMs caused morphological and functional alterations to the cells. Infection with SARS-CoV-2 at a high MOI caused loss of beating and increased apoptosis, with the contractile dysfunction demonstrated in both 2D hPSC-CMs monolayers (Sharma et al., 2020) and EHTs (Marchiano et al., 2020). However, even when the hPSC-CMs were infected at a very low MOI, sarcomeric fragmentation as well as loss of nuclear DNA was observed, including in adjacent apparently uninfected hPSC-CMs (Pérez-Bermejo et al., 2020). Whether these bystander effects are an artifact of the model or whether even minimal exposure to the virus directly damages the heart in patients, as has been suggested (Guo et al., 2020), requires further examination. RNA-sequencing analysis also revealed a wide range of transcriptional changes upon infection, including in structural and contractile genes and in pathways related to immune regulation. Moreover, autopsy specimens from COVID-19 patients confirmed these *in vitro* phenotypes (sarcomeric disruption, myofibrillar anomalies, and loss of nuclear DNA), validating the suitability of hPSC-CMs as a model for studying pathogenic viral infections of the human cardiovascular system.

Apart from these contractile and structural alterations, SARS-CoV-2 also appears to alter the electrophysiological properties of hPSC-CMs (Marchiano et al., 2020). The field potential duration (FPD), which can be measured *in vitro* using a multi-electrode array, correlates to the QT interval measured using an electrocardiogram in patients, with prolongation in both systems being a pro-arrhythmogenic indicator. SARS-CoV-2 infection of hiPSC-CMs resulted in lower depolarization spike amplitude, FPD prolongation, and decreased electrical conduction velocity, although whether the virus is directly responsible for arrhythmias observed in COVID-19 patients or they are a consequence of myocardial injury needs further exploration.

**Figure 2:**
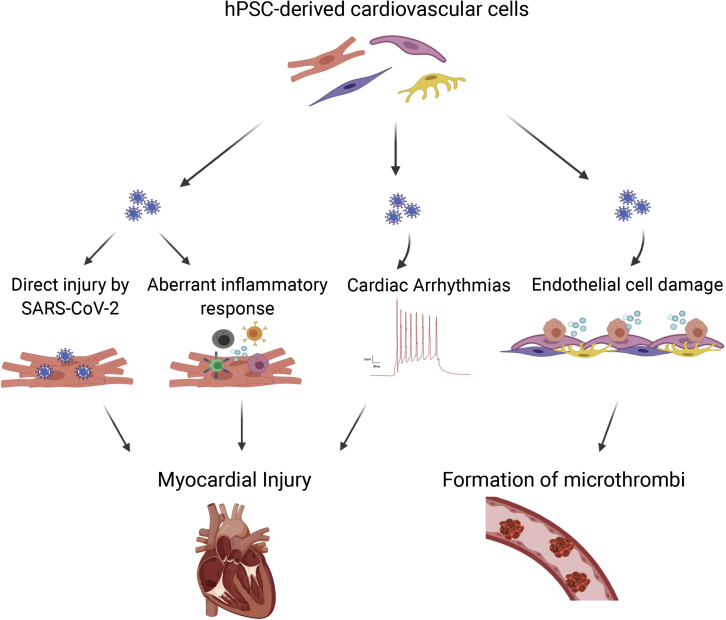
Overview of the Use of hPSC-Derived Cardiovascular Cells to Study the Effects of SARS-CoV-2 Infection in the Heart The mechanisms leading to myocardial injury can be studied by looking at the effects of (in)direct viral interaction with cardiomyocytes or endothelial cells, as well as the role an aberrant inflammatory response has in contributing to tissue damage and the formation of microthrombi in COVID-19 patients.

Indeed, arrhythmias in COVID-19 patients could also be caused by fever, sepsis, hypoxia, or electrolyte imbalances or even be drug induced. As SARS-CoV-2 can affect the renin-angiotensin-aldosterone system (RAAS), the resulting hypokalemia could indirectly disrupt heart rhythm (Bansal, 2020). Monitoring the effect of infection on the patient's heart is thus critical to predict and avoid lethal arrhythmias in COVID-19 patients (Figure 2). In addition, interaction of the S protein with ACE2 upregulates the Ras-ERK-AP-1 pathway leading to activation of pro-fibrotic factors such as the C-C motif chemokine ligand 2 (CCL2) (Chen et al., 2010). This can also contribute to cardiac injury and may subsequently cause the fibrosis associated with disease manifestation. Multicellular hPSC-derived cardiac tissues composed of multiple cell populations present in the heart enable intercellular cross talk to occur (Giacomelli et al., 2020), and represent new models for studying the cause of fibrosis in COVID-19 patients.

In addition to the acute adverse effects of SARS-CoV-2 infection and myocardial injury observed in fatal cases, COVID-19 can also cause damage to the heart through other mechanisms that might contribute to longer-term effects after infection. These include, for example, thrombotic microangiopathy and venous thrombosis, indicated by the elevated thrombogenic biomarker D-dimer observed in COVID-19 patients (Geng et al., 2020). How this pathology develops is unclear, but it is probably multifactorial, with contributions from the hyperinflammatory response seen in COVID-19 patients and damage to the ECs caused by SAR-CoV-2. Thus, ongoing studies are aimed at shedding light into which mechanisms are most likely, since they will determine potential treatment strategies. Here, too, hPSC-derived cardiovascular cells provide an accessible system to study thrombosis development (for example, in combination with microfluidic devices that mimic perfused vasculature), metabolic activity, and dysregulation upon SARS-CoV-2 infection over time.

The long-term cardiovascular effects of COVID-19 are a very important aspect to consider. Studies on patients who recovered from SARS, as well as from other respiratory tract infections, have shown dysregulated lipid and glucose metabolism (Corrales-medina et al., 2015; Wu et al., 2017). Furthermore, persistent cardiovascular damage has been reported in patients that have recovered from COVID-19. In a recent study involving 100 patients with a median age of 49, 78% of recovered patients had persistent elevated levels of troponin I 2–3 months after infection, while 60% had ongoing myocardial inflammation (Puntmann et al., 2020). Importantly, the effects were independent of COVID-19 severity. Although the findings still need to be replicated in a larger cohort and it remains to be determined if the abnormalities persist more long term, the study raises further concerns about patients whose symptoms of the disease were mild at the time of the infection. Whether these subsequent cardiovascular abnormalities are directly due to the infection, the resulting systemic inflammatory activity, or the therapeutic compounds administered also remains unclear. Again, here, hPSC-derived cardiovascular models will provide a useful tool to study the long-term effects of the infection, help answer these key questions, and assist in determining treatments that recovering COVID-19 patients may need post-infection.

#### Immune Response and the Cytokine Storm

It is thought that the main contributor in COVID-19 pathogenesis is the excessive and uncontrollable cytokine production that results from the infection, referred to as a cytokine storm (Tang et al., 2020). Increased levels of pro-inflammatory cytokines, including IL-6, IL-10, and TNF-α, have been found in COVID-19 patients and are associated with more severe disease progression (Huang et al., 2020; Zhou et al., 2020a). Specifically, autopsy reports have shown inflammatory infiltrates composed of macrophages and CD4^+^ T cells in cardiac tissue, indicating that an aberrant immune response in the heart can contribute to severe disease and cardiac injury (Bansal, 2020; Zheng et al., 2020).

It is known that inflammatory cytokines are toxic for CMs and ECs (Clyne et al., 2008; Jarrah et al., 2018), and while this is a possibility in COVID-19 patients, the exact mechanisms through which an aberrant immune response contributes to cardiac injury is unknown. Moreover, the specific immune cells that mediate cardiac injury are unknown. It is thus of utmost importance to determine which cell types present in the heart or infiltrate express the inflammatory cytokines for better understanding of the immune response following infection. The ability to generate multicellular *in vitro* models could provide suitable physiological models for investigating not only how the disease affects the various cardiovascular cells types, but also potentially how inflammatory responses can lead to further heart damage via direct and indirect mechanisms. A preprint using human cardiac organoids composed of hiPSC-CMs and hiPSC-ECs reported that inflammatory mediators such as IFN-γ and IL-1β, combined with dsRNA, caused severe diastolic dysfunction (Mills et al., 2020), thereby indicating that the induced cytokine storm might play a key role in cardiac damage in COVID-19 patients. A separate report further validated that SARS-CoV-2 induces innate immune activation and interferon response in hPSC-CMs (Marchiano et al., 2020). Further development of multicellular 3D hiPSC-derived cardiac models to incorporate either immune cells (e.g., macrophages, natural killer cells, or T cells) or specific cell types infected with SARS-CoV-2 will allow the immune and inflammatory response in the heart to be studied by examining the interaction and cross talk between multiple cell types. This could enable the innate immune responses at the early stage of infection to be further investigated, as well as any long-term effects.

The important role that the cytokine storm plays in COVID-19 means that the use of monoclonal antibodies as therapeutics might be both beneficial and effective. Here, too, multicellular hPSC-derived systems will provide useful tools for screening various antibodies against interleukins and interleukin receptors prior to their use in clinical trials. Overall, these approaches can facilitate not only further understanding of the role of the immune response in the heart but also the development of new therapeutics targeting the cytokine storm and mitigating severe cardiovascular damage.

#### Pre-existing Cardiovascular Co-morbidities

As observed with both SARS and MERS, COVID-19 patients with pre-existing co-morbidities, including cardiovascular conditions such as hypertension, coronary heart disease, or acute coronary syndrome, are more likely to develop severe symptoms and present with poorer prognosis (Wang et al., 2020a). A number of reasons are believed to contribute to this phenomenon. First, increased ACE2 expression is observed after myocardial infarction and in response to treatment of hypertension, potentially making patients more vulnerable to COVID-19 (Burrell et al., 2005). Moreover, the dysregulated immune system observed in patients with hypertension could further exacerbate the negative effects of COVID-19 (Guzik et al., 2020). However, numerous other factors also could contribute to the increased susceptibility of these patients. Thus, it is vital to further understand the interplay of existing co-morbidities with SARS-CoV-2 infection. This will provide information on how treatment options for COVID-19 patients could be tailored based on pre-existing CVD co-morbidities. Recent advances in 3D culture systems that include hPSC-CMs are now leading to the development of models for complex diseases such as ischemia-induced injury, polygenic hypertension, fibrosis, and atherosclerosis (Simon and Masters, 2020). Such models also could be utilized to study the interaction of SARS-CoV-2 with pre-existing injury.

Another important aspect to consider when treating COVID-19 patients with co-morbidities is what current medication they have been prescribed. One example is ACE inhibitors (ACEis) and angiotensin receptor blockers (ARBs) taken by patients with hypertension. These inhibitors can increase ACE2 levels and thus potentially could result in a more severe disease response in COVID-19 patients. However, whether these inhibitors play a role in COVID-19 manifestation remains unresolved, as contradictory evidence has not facilitated a consensus on whether they should continue to be taken by the patient if infected with SARS-CoV-2. Here, too, hPSC-derived models could assist in answering this question. For example, hPSC-derived cardiovascular cells could be treated with these antihypertensive drugs and ACE2 expression, as well as the effects of these inhibitors on the RAAS, examined in the CMs and ECs. It is also essential to investigate downstream signaling pathway activity in response to SARS-CoV-2 infection, to identify whether indeed there is a pathological phenotype compared with untreated conditions. If ACEis and/or ARBs contribute to more severe disease manifestation, then alternative antihypertensive drugs, such as beta blockers, or non-dihydropyridine calcium antagonists, such as verapamil, might be alternative medications that could be taken by these patients, as discontinuing such treatments is likely to exacerbate hypertension. Overall, it is critical to determine the interplay of antihypertensive drugs with SARS-CoV-2 infection in order to improve patient management and develop more personalized treatments depending on the condition and co-morbidities existing in each individual.

### COVID-19 Therapies and Potential Cardiovascular Interactions

Current strategies for treating COVID-19 aim to either repurpose drugs approved for treating other diseases or use recently developed medicines that have been granted approval for compassionate use. However, many of these drugs, which include several conventionally used to treat other viral infections, can have harmful side effects. For example, potential candidates for COVID-19 treatment include or have included the antimalarial drug chloroquine/hydroxychloroquine, the antibacterial drug azithromycin, and the immunosuppressant azathioprine (Gautret et al., 2020; Wang et al., 2020c; Yao et al., 2020). These drugs are known to prolong the cardiac QT interval (Hancox et al., 2013; Roden et al., 2020) and could thus lead to life-threatening arrhythmias. These potential side effects might be even more severe in cases where the drugs are used in combination with other medications taken by COVID-19 patients, such as antibiotics and antipyretics, leading to a reduced repolarization reserve (Li and Fu, 2013). Further prolongation of QT interval and heart rhythm compilations in these patients likely would have adverse effects and may lead to increased risk of death.

Clinical studies involving COVID-19 patients have indeed confirmed that the possibility of cardiac arrest was more likely in patients treated with hydroxychloroquine and azithromycin than in patients not treated with these drugs (Zhang et al., 2020). In addition, the extent of QTc prolongation was greater in COVID-19 patients treated with this combination of medications compared with a previous study in healthy volunteers (Chorin et al., 2020). Whether this is due to the infection or other patient-specific co-morbidities is worthy of follow-up and could be performed using some of the hPSC-cardiac models already described.

The relatively new antiviral drug remdesivir has been shown to result in faster clinical recovery in COVID-19 patients (Beigel et al., 2020, Wang et al., 2020ee). Although no significant adverse cardiovascular effects have been reported, clinical pharmacological data as well as drug interaction studies with this compound or its active metabolite are limited. As well, the enzymes involved in the metabolism of remdesivir and its metabolites are not clearly identified. Again, here, hPSC-differentiated cells could be leveraged to rapidly provide human-based data. In particular, hPSC-derived cardiovascular models could accelerate preclinical safety and toxicity assessments in comparison to animal studies and facilitate more efficient planning of clinical trials.

The utility of hPSC-derived cardiovascular cells for compound screening is unambiguous (Blinova et al., 2018; Saleem et al., 2020), and they will likely be an invaluable tool for assessing the suitability of novel candidate drugs for COVID-19 treatment. This was demonstrated in a recent preprint that utilized hPSC-CMs to screen for the antiviral effect of different protein kinase inhibitors currently in clinical trials (Garcia et al., 2020). Specifically, berzosertib, an inhibitor of ATR kinase involved in DNA-damage response, demonstrated potent antiviral activity, validating the original results of the study on non-human cell lines. Moreover, the use of human cardiac organoids led to the identification of the epigenetic regulator BRD4 as a therapeutic target of the cytokine storm (Mills et al., 2020). In the study the authors tested various BRD inhibitors in human cardiac organoids and identified that the inhibitor INCB054329 was capable of preventing cardiac cytokine storm-induced diastolic dysfunction. These and future studies using hPSC-cardiovascular cells will be undoubtedly valuable in paving the way for the potential use of this drug in COVID-19 patients.

Another aspect where hPSC-derived CMs will play an important role is in assessing whether these candidate drugs have a greater risk of causing QT prolongation and arrhythmias in patients with genetic cardiac disorders such as long QT syndrome (LQTS). Naturally, such patients are typically excluded from clinical trials. However, with congenital LQTS occurring at a frequency of ∼1 in 2,000 individuals, it is clear that, given SARS-CoV-2 has infected >30 million people, LQTS patients are also requiring treatment. The numerous patient-derived hPSC disease models now available for genetic cardiac disorders (van den Brink et al., 2019; Protze et al., 2019) provide a unique opportunity to determine rapidly which cardiac diseases and mutations are more sensitive to the use of these novel drugs. Such studies can be used in combination with SARS-CoV-2 infection and/or multiple drug treatments, modeling a clinical scenario where patients with variable genetic backgrounds undergo the same treatment. Using hPSC-derived models in such a way to confirm that a particular treatment does not increase the risk of drug-induced sudden cardiac death potentially could allow subsets of patients to receive promising new treatments that they otherwise might not have received. Similarly, in instances where the hPSC disease models show a more severe phenotype due to the treatment, additional countermeasures to mitigate the risk in those patients can be included.

In summary, it is preferable to test the effects of repurposed and novel drugs on cardiac toxicity prior to prescribing them to treat COVID-19. Indeed, hPSC-derived CMs and high-throughput techniques to screen for drug toxicity provide a tool that can quickly provide valuable information on which repurposed or novel therapeutics are good candidates.

### Genetic Prediction of COVID-19 Risk and Severity

Genetic factors and common genetic variants play a major role in disease susceptibility and progression in the general population. The variable COVID-19 severity observed among individuals of the same age or gender, or even within a society, could in part be attributed to the predisposition given by genetic factors. The versatility of hPSCs, including the ability to genetically modify them, means they are a valuable tool for validating and discovering genetic variants predicted as risk factors for various diseases. A recent genome-wide association study (GWAS) of COVID-19 patients revealed a gene cluster on the genomic region 3p21.31 that confers increased susceptibility to the disease (Ellinghaus et al., 2020). This cluster includes a number of genes that can be directly implicated in COVID-19 progression. For example, the gene *SLC6A20* encodes the sodium-imino acid (proline) transporter 1 (SIT1), known to functionally interact with ACE2. In addition, this region contains genes encoding chemokine receptors, which could be highly relevant for the differential immune responses seen among patients. Moreover, single-nucleotide polymorphisms in *ACE2* as well as *TMPRSS2* have been identified, which could contribute to the variable disease severity observed in patients (Asselta et al., 2020; Hussain et al., 2020).

To functionally validate the potential role of specific genetic variants in COVID-19 pathology, and in particular their effects in the heart, panels of hPSC-cardiovascular cells carrying these genetic variants could be generated and used to establish how they contribute to the variable disease severity. Furthermore, candidate genes from GWASs could be investigated by gene knockout and overexpression studies, or by introducing specific variants into the hPSCs, which are then subsequently differentiated to study their effects in cardiovascular cells. Such an approach is expected to yield beneficial outcomes in three ways: (1) genetic risk factors that could provide a diagnostic and prognostic tool for risk stratification in the clinic could be identified and validated, (2) determining which COVID-19 patients are at higher risk of developing severe disease will better inform clinicians on treatment decisions, and (3) hPSC models carrying certain genetic variants will allow the further study of how a particular genotype leads to a more severe pathology and possibly contribute to the identification of novel therapeutic targets.

### Conclusions and Perspectives

**Figure 3:**
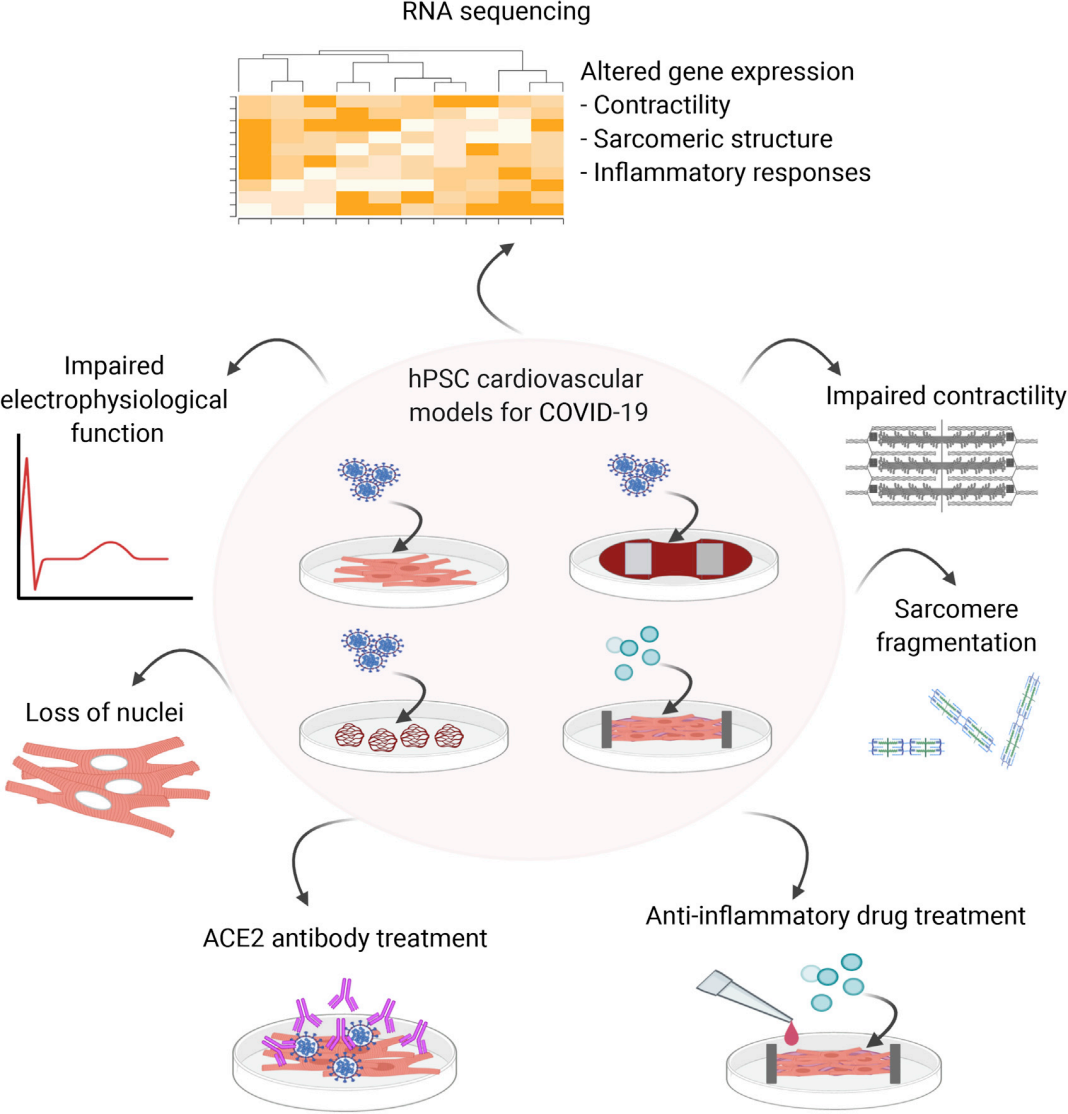
Overview of the Current Applications Reported That Have Used hPSC-Derived Cardiovascular Models to Investigate Pathological Aspects of COVID-19 hPSC-derived CMs, vascular organoids, and EHTs have been either infected with SARS-CoV-2 or exposed to conditions mimicking a cytokine storm. Findings include impaired CM contractility and electrophysiology, sarcomeric fragmentation, and loss of nuclei. Gene expression analysis also has indicated dysregulated sarcomeric contractile and structural transcription, as well as activation of innate inflammatory responses. The models have also been applied in antibody and compound screens for identifying potential therapeutic treatments.

The potential of hPSCs for modeling disease is well recognized, and has already had a significant societal impact through drug screening studies and in human preclinical “trials” for a number of diseases. More specifically, hPSC-derived cardiac models have provided information on fundamental aspects of human biology and development, as well as numerous cardiac diseases. These advances in our understanding of human cardiovascular pathology and the identification of numerous therapeutic targets brings the scientific community one step closer to regenerative and personalized medicine. As such, these models are already starting to be used to uncover aspects of COVID-19 pathology and to develop treatments for COVID-19, thus having an impact on our ability to overcome the current pandemic (Figure 3). The investigation of virus-human interactions is now a priority for the scientific community. This work will provide valuable knowledge not only regarding COVID-19, but also for any future (new) viral infections. In summary, existing and newly developed hPSC cardiac models, combined with cellular, biochemical, molecular, and genome-wide studies, will contribute to revealing key mechanisms and therapeutic avenues for treating cardiovascular damage caused by COVID-19.

## Author Contributions

L.Y. performed the literature search and wrote the manuscript. R.P.D. and C.L.M. revised and wrote the manuscript. All authors reviewed the manuscript and approved the final version.

## Conflicts of Interest

C.L.M. is a co-founder of Ncardia BV.
